# Sodium and potassium intake estimated using two methods in the Brazilian Longitudinal Study of Adult Health (ELSA-Brasil): Consumo de sódio e potássio estimado por dois métodos no Estudo Longitudinal de Saúde do Adulto (ELSA-Brasil)

**DOI:** 10.1590/1516-3180.2015.01233108

**Published:** 2015-04-14

**Authors:** Taísa Sabrina Silva Pereira, Isabela Judith Martins Benseñor, Jorge Gustavo Velásquez Meléndez, Carolina Perim de Faria, Nágela Valadão Cade, José Geraldo Mill, Maria del Carmen Bisi Molina

**Affiliations:** I MSc. Doctoral Student of Public Health, Universidade Federal do Espírito Santo (UFES), Vitória, Espírito Santo, Brazil.; II MD, PhD. Associate Professor, Department of Internal Medicine, School of Medicine, Universidade de São Paulo (USP), São Paulo, Brazil.; III PhD. Professor in the Department of Maternal and Child Nursing and Public Health, School of Nursing, Universidade Federal de Minas Gerais (UFMG), Belo Horizonte, Minas Gerais, Brazil.; IV PhD. Assistant Professor, Department of Integrated Health Education, Universidade Federal do Espírito Santo (UFES), Vitória, Espírito Santo, Brazil.; V PhD. Associate Professor, Department of Nursing, Universidade Federal do Espírito Santo (UFES), Vitória, Espírito Santo, Brazil.; VI PhD. Titular Professor, Department of Physiology, Universidade Federal do Espírito Santo (UFES), Vitória, Espírito Santo, Brazil.; VII PhD. Associate Professor, Department of Integrated Health Education, Universidade Federal do Espírito Santo (UFES), Vitória, Espírito Santo, Brazil.

**Keywords:** Epidemiologic studies, Electrolytes, Biological markers, Diet, Urine specimen collection.

## Abstract

**CONTEXT AND OBJECTIVE:**

Sodium and potassium intake from different food sources is an important issue regarding cardiovascular physiology. Epidemiological assessment of the intake of these electrolytes intake is done through food frequency questionnaires or urinary excretion measurements. Our aim was to compare these methods using a sample of Brazilian civil servants.

**DESIGN AND SETTING:**

Cross-sectional baseline evaluation from the Brazilian Longitudinal Study of Adult Health.

**METHODS:**

Sodium and potassium intake was obtained using two methods: a semi-quantitative questionnaire including 114 food items; and overnight 12-hour urinary excretion measurement. Sodium and potassium estimates obtained through the questionnaire were adjusted for energy intake using the residual method. Urinary excretion measurements were considered valid if they met three adequacy criteria: collection time, volume and total creatinine excretion. Mean nutrients were estimated, and Spearman correlations were calculated. Sodium and potassium intake was categorized into quintiles, and weighted kappa coefficients and percentage agreement were calculated. The significance level for all tests was 0.05.

**RESULTS:**

Data from 15,105 participants were analyzed, and significant differences between mean intakes of sodium (questionnaire: 4.5 ± 1.7 g; urine: 4.2 ± 2.1 g) and potassium (questionnaire: 4.7 ± 1.8 g; urine: 2.4 ± 1 g) were found. Weak agreement was found for sodium (K = 0.18) and potassium (K = 0.16). The percentage disagreement between methods ranged from 41.8 to 44.5%, while exact concordance ranged from 22.1% to 23.9%.

**CONCLUSIONS:**

The agreement between the food frequency questionnaire and urinary excretion measurements for assessment of sodium and potassium intakes was modest.

## RESUMO

**CONTEXTO E OBJETIVO:**

O consumo de sódio e potássio de diferentes fontes alimentares é uma questão importante para a fisiologia cardiovascular. A avaliação epidemiológica do consumo desses eletrólitos é feita pelo questionário de frequência alimentar ou pela excreção urinária. O objetivo deste estudo é comparar esses métodos em uma amostra de servidores públicos brasileiros.

**TIPO DE ESTUDO E LOCAL:**

Avaliação transversal da linha de base do Estudo Longitudinal de Saúde do Adulto.

**MÉTODOS:**

O consumo de sódio e potássio foi obtido por dois métodos: questionário semi-quantitativo com 114 itens alimentares e excreção urinária de 12 horas noturnas. Estimativas de sódio e potássio obtidas pelo questionário foram ajustadas pela energia utilizando o método residual. A excreção urinária foi considerada válida se atendesse a três critérios: tempo de coleta, volume e excreção total de creatinina adequados. Foram estimadas médias dos nutrientes e calculada a correlação de Spearman. O consumo de sódio e potássio foi categorizado em quintis e foram calculados o *kappa* ponderado e o percentual de concordância. O nível de significância para todos os testes foi de 0,05.

**RESULTADOS:**

Foram analisados dados de 15,105 participantes e encontradas diferenças significativas entre médias de sódio (questionário: 4,5 ± 1,7 g; urina: 4,2 ± 2,1 g) e potássio (questionário: 4,7 ± 1,8 g; urina: 2,4 ± 1 g). Foi encontrada fraca concordância para sódio (K = 0,18) e potássio (K = 0,16). Percentuais de discordância entre métodos variaram de 41,8-44,5%; concordâncias exatas de 22,1-23,9%.

**CONCLUSÃO:**

A concordância entre o questionário de frequência alimentar e excreção urinária para avaliação do consumo de sódio e potássio foi modesta.

## INTRODUCTION

Sodium and potassium intake is vital for human life, but excess of sodium and insufficiency of potassium are associated with adverse health outcomes.^1^ ^2^ ^3^ A high intake of sodium indicates a diet rich in manufactured foods, since the food industry uses it to enhance the flavor and prolong the shelf life of processed foods.^4^ Potassium is considered to be a marker of a healthy diet, since it is a nutrient found in fruits and vegetables.^5^

Evaluating diet in population-based studies is a challenge, although there are several methods available, such as food frequency questionnaires (FFQs), food registers, 24-hour dietary recalls and intake biomarkers. Each method has its advantages and also its limitations.^6^ Among the methods used to evaluate diet, FFQs are frequently used in epidemiological studies. They are less expensive than other methods for evaluating food intake, and they enable correlation between diet and occurrences of diseases.^6^ ^7^ ^8^ However, they have limitations, such as the need to use composition tables, which may change from one country to another, thus making comparisons difficult. Also, FFQs often do not include regional preparations and manufactured foods produced in specific places, and can overestimate dietary intake. 

Moreover, evaluations on food intake may become biased by several factors, such as memory errors, inaccurate estimation of portion sizes and frequency of consumption, and adequacy of the lists in terms of the food items included.^6^ In the case of measurement of sodium intake, there are some specific problems due to the wide variability between individuals, in relation to the salt added to food preparations and at meals. Since the food recipe used in the FFQ is standardized, some of this interpersonal variability is lost. 

In the case of sodium and potassium, it is possible to measure 24-hour urinary excretion, which is the gold standard for their measurement.^9^ ^10^ However, the logistics of collecting 24-hour urine samples are not so simple in studies with large samples. As an alternative, 12-hour urine samples collected overnight can simplify the logistics, since it is easier for the participant. This strategy has been validated for the adult population.^11^

## OBJECTIVE

The aim of this study was to compare sodium and potassium intake measurements, estimated through a food frequency questionnaire and through 12-hour urinary excretion, among participants at the baseline of the Brazilian Longitudinal Study of Adult Health (ELSA-Brasil).

## METHODS

Data were collected from all participants at the baseline of the ELSA-Brasil study, which was a prospective cohort study on 15,105 active or retired civil servants of both sexes, aged between 35 and 74 years, working at the six public higher education institutions at which ELSA-Brasil was conducted.^12^ ELSA-Brasil was approved by the Research Ethics Committees of the six institutions. The participants attended one of the six research centers on the scheduled date for clinical and laboratory tests to be conducted, and for questionnaires to be answered during interviews.^12^

Overnight 12-hour urine collections were used in ELSA-Brasil to estimate electrolyte excretion levels (sodium, potassium and calcium), as previously described by Mill et al.^11^ While scheduling the examinations, the participants received verbal and written information on how to collect their urine, and were given a two-liter plastic bottle for this purpose. The participants were instructed to collect their urine samples between 7 pm and 7 am on the following morning, and to take note of the exact start and end time of the collection, as well as any losses. The notes and the urine collected were brought in by each participant on the day of the examinations. Aliquots of urine were sent to the ELSA-Brasil Central Laboratory for measurements on creatinine level (Jaffé method) and on sodium and potassium (ion-selective electrode method).

A 12-hour urine collection was considered valid if it simultaneously met three criteria: total collection interval of between 10 and 14 hours; collected volume of 250 ml or more; and total creatinine excretion, corrected for body weight, of between 14.4 and 33.6 mg/kg for males and 10.8 and 25.2 mg/kg for females.^13^ As proposed by Mill et al.,^11^ data from the overnight 12-hour urinary excretion were used to estimate the 24-hour sodium and potassium intake.^11^ Salt intake was calculated based on the estimated 24-hour urinary sodium excretion, assuming that all sodium was consumed in the form of sodium chloride.

The FFQ used in the study was a semi-quantitative questionnaire with 114 food items, which had the aim of assessing regular food consumption over the last twelve months.^14^ It had been validated for the ELSA-Brasil sample.^15^ The nutritional composition of each food item included in the FFQ had been estimated using the database of the Nutrition Data System for Research, version 2010.^16^ For one food item (cassava flour) that could not be found in this database, the nutritional composition was extracted from the Brazilian Table of Food Composition.^17^ The nutrient composition of regional preparations used in ELSA-Brasil was calculated based on recipes provided by technical research publications and teaching institutions, as previously published.^15^

After a preliminary analysis on the data from the FFQ, implausible values were replaced by values corresponding to the 99^th^ percentile of distribution of that specific food item. Also, when the participant reported seasonal consumption of an item, the daily intake of that item was multiplied by 0.25.

In the present analysis, we followed the guidelines presented by Cobb et al. in 2014.^18^ These authors recommended excluding participants who reported following a diet with sodium restriction, those with cardiovascular disease or diabetes^18^ and those who reported a total daily calorie intake of ≤ 500 kcal or > 6000 kcal.^19^

### Statistical analysis

The sodium and potassium intake was adjusted for total energy consumption using the residual method.^20^ The Kolmogorov-Smirnov test was used to test the normality of the variables. Means and standard deviations of sodium and potassium values were estimated for both methods (FFQ and urinary excretion). These estimates were then compared using the Mann-Whitney test.

To assess the degree of agreement between the two methods, the estimated sodium and potassium intake was categorized into quintiles. Then, the percentages of exact agreement (same quintile), adjacent agreement (adjacent quintiles) and disagreement (opposite quintiles) were calculated. Weighted kappa coefficients were used to evaluate the concordance between quintiles. Spearman's correlation was used to evaluate the relationship between the two methods. Correlation coefficients can range from -1 to +1 and can be categorized as weak (r < 0.3), moderate (r = 0.3-0.7) or strong (r > 0.7).^9^ The significance level for all tests was 0.05. The data were analyzed using the Statistical Package for the Social Sciences (SPSS), version 17.0.

## RESULTS

**Figure 1:**
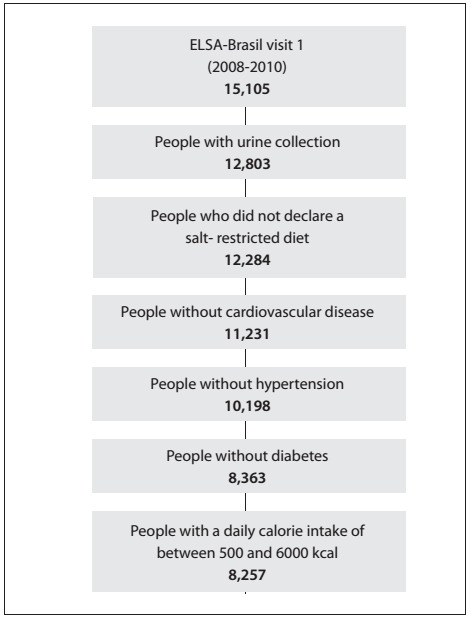
Defiition of the sample of participants of the ELSABrasil, 2008-2010.

Among the 15,105 participants, the following were excluded: those for whom the urine collection was not validated; those who reported following sodium restriction diets; those with previous cardiovascular diseases; those with a diagnosis diabetes and hypertension at the baseline; and those whose total energy intake was less than 500 kcal or higher than 6000 kcal. Thus, 8,257 participants remained in the analysis (Figure 1).

These 8,257 participants presented a mean age of 52 ± 9 years; 43.2% (n = 3,566) were male and 56.8% (n = 4,691) were female. Table 1 presents a comparison between estimated means for sodium and potassium intake: both crude and adjusted for total energy, using the FFQ and the estimated salt intake. Significant differences were found between the estimated mean values (in grams) of sodium in urine (4.2 ± 2.1), unadjusted FFQ (4.5 ± 1.7) and energy-adjusted FFQ (4.3 ± 0.7) (P < 0.001). Significant differences were also observed between the average potassium intake (urine: 2.4 ± 1; unadjusted FFQ: 4.7 ± 1.8; and adjusted FFQ: 4.5 ± 1.0; P < 0.001) and the estimated salt intake (urine: 10.5 ± 5.2; unadjusted FFQ: 11.2 ± 4.2; and adjusted FFQ: 10.6 ± 1.7; P < 0.001).

**Table 1:**
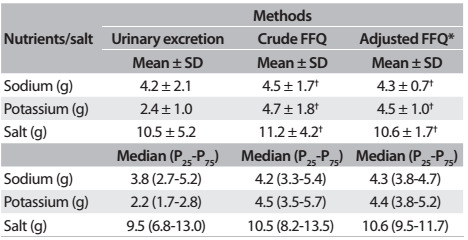
Sodium, potassium and salt intake, estimated through urinary excretion and crude and adjusted food frequency questionnaire (FFQ) data, among participants in ELSA-Brasil, 2008-2010

Table 2 presents the exact agreement, adjacent agreement and disagreement of the estimated quintiles of sodium and potassium using urinary excretion and FFQ (both crude and adjusted for energy), as well as the weighted kappa coefficient for each nutrient. The percentage disagreement for sodium estimates was 41.8% (unadjusted) and 44.5% (adjusted). For potassium, the percentage disagreement was 42.6% (unadjusted) and 42.8% (adjusted). The calculated exact agreement (same quintile) was 23.4% for unadjusted sodium and 22.1% for adjusted sodium, and it was 23.9% for unadjusted potassium and 23.5% for adjusted potassium. The weighted kappa coefficient for unadjusted nutrients was 0.18 (sodium) and 0.16 (potassium).

**Table 2:**
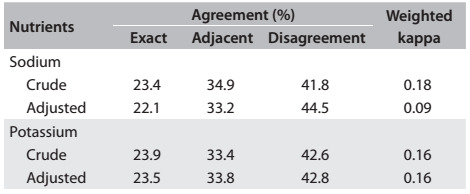
Correlation of estimated sodium and potassium levels between 12-hour urinary excretion measurement and the food frequency questionnaire among participants of ELSA-Brasil, 2008-2010

**Figure 2:**
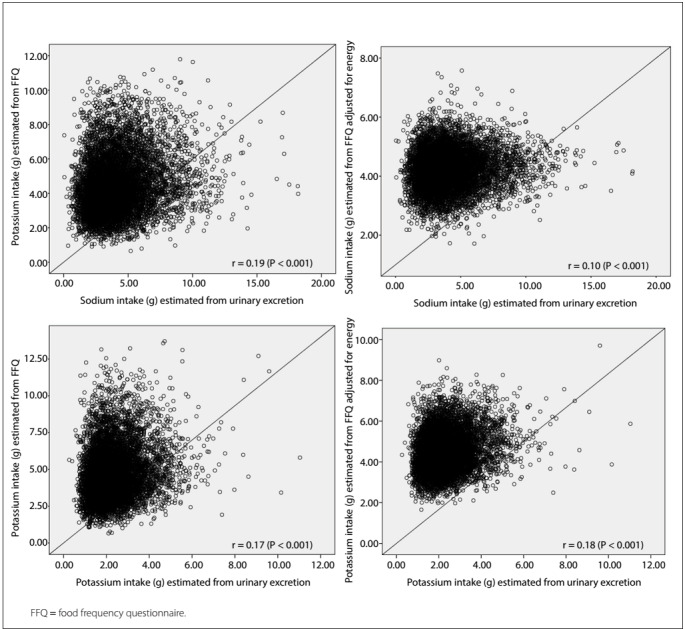
Correlation of estimated consumption of sodium and potassium between the methods of urinary excretion and food frequency questionnaire (FFQ), expressed as crude values and values adjusted for energy, among participants of ELSA-Brasil, 2008-2010.

Figure 2 presents the Spearman correlation coefficients for the estimates of sodium and potassium intake, as measured through overnight 12-hour urinary excretion, and through unadjusted and energy-adjusted FFQs. The correlations were less than 0.3 and were thus considered weak.^1^

## DISCUSSION

In this study, the estimated sodium and potassium intake, as measured using the FFQ, presented modest correlations with 12-hour urinary excretion measurements.

The estimated means for sodium and potassium intake measured using the FFQ in this study were different from the results of the Brazilian Household Budget Survey (Pesquisa de Orçamento Familiar),^21^ which collected data in Brazil between 2008 and 2009. Food records from a subsample of houses were assessed, and the sodium and potassium intake was seen to be different from our results.^21^ Two possible explanations for these differences are the method used to measure sodium and potassium intake, and the different age strata between the two studies. 

In a study on hypertensive individuals over 50 years of age, Dallepiane et al.^22^ compared two methods for evaluating sodium intake: a questionnaire with 21 food items that considered important sources of sodium; and 24-hour urinary excretion. The correlations between the two methods were low and not significant, and the authors concluded that despite the advantages of the questionnaire, it could not be used for this purpose.^22^ In a validation study on a FFQ using biomarkers, Sauvageot et al.^23^ also reported weak correlations of sodium between the two methods. Ferreira-Sae et al.^24^ validated a FFQ with 50 food items that were sources of sodium intake, among hypertensive individuals. They did not report any significant correlations between FFQ and 24-hour urinary excretion.^24^ In a study in Porto Alegre, in southern Brazil, Micheli and Rosa^25^ did not find good agreement between the food register and 12-hour urinary sodium excretion. 

High amounts of sodium are generally found in sausages and other manufactured products. Salt added in the preparation of meals is another important source of sodium. Therefore, the weak correlation between the FFQ and overnight urinary sodium may be explained by high interpersonal variability in urinary sodium. 

Urinary excretion of sodium and potassium has been presented as a more accurate method for measuring the intake of these nutrients, although its use in large studies has important limitations with regard to urine collection done by the study participants. However, it does not identify the sources of foods associated with this high intake. Through using both the FFQ and 24-hour urinary excretion, we obtained measurements of the intake as well as identifying the dietary sources of sodium and potassium. One potential explanation for the differences in nutrient estimates obtained through the two methods used may be related to the intrinsic methodological aspects of the measurement technique: the FFQ reflects the habitual consumption over the last twelve months, while the figures presented through urinary excretion reflect the participant's food consumption over a period closer to the time of sample collection.

Application of statistical methods helps minimize and correct for the wide variability that is characteristic of diets. In this study, after adjustment of nutrients for energy intake, different behaviors were observed, namely, an increase in the estimated intake of potassium and a decrease in the intake of sodium. A similar result was also found in another study in which nutrients were adjusted for energy intake.^5^ The adjustment for energy intake may increase the correlation coefficient, as occurred with potassium in our data. If the nutrient intake is related to the energy intake, it may decrease when the variability of a specific nutrient intake is related to systematic errors of under or overestimation in the reporting of food consumption, as probably occurred with sodium in this analysis.^9^

Although we used the database of the Nutrition Data System for Research, which would make it possible to discriminate the amount of salt and condiments added to the food preparations of each participant, this was not done in our study, because a FFQ with 114 items was used, and because of the high number of participants (15,105). The recipe of the preparations was the same for all participants, since the FFQ focuses on frequency and portion size. All of these points may explain the finding of a weak correlation between the two methods. 

Our study has some strengths and limitations. It included a large sample of participants and used a FFQ built especially for the study. However, urinary excretion data were collected only once. We followed the same strategy as that of Cobb et al.,^18^ which has now become a guideline for studies that measure sodium intake. This limitation is observed in all major epidemiological studies, in which the complex logistics of sample collection seem to limit measurements to once only. Another limitation is that we collected overnight 12-hour and not 24-hour urinary excretion measurements. Since the participants remained in the research center for approximately 6 hours in order to attend the interviews and undergo several tests, they missed one day of work. It is complicated to collect 24-hour urine at work. Had we chosen the option of 24-hour collection, we would have had to ask the participants to miss another day of work, thereby decreasing the adhesion to the study. 

Finally, this modest agreement may be explained by the different focuses between these two methods. Urinary excretion is an accurate tool for quantifying sodium intake at a specific point in time, while the FFQ focuses on the estimated usual intake of a nutrient over the last year. Although the FFQ is very useful in epidemiological studies, for classifying individuals according to their intake levels, it has important limitations, especially in the case of sodium, which relate to the difficulty in capturing the interpersonal variability in sodium intake observed in food preparation and at meals.

## CONCLUSIONS

Our results showed that the agreement between a food frequency questionnaire and urinary excretion measurements for assessment of sodium and potassium intake was modest.
